# *Schistosoma mansoni* Infections Are Associated With Fecal Calprotectin Markers of Gut Inflammation After Accounting for HIV, Hepatitis B, and Malaria

**DOI:** 10.1093/infdis/jiag094

**Published:** 2026-04-29

**Authors:** Lauren Wilburn, Hanh Lan Bui, Paul Akampurira, Reagan Ddamba, David W Oguttu, Betty Nabatte, Narcis B Kabatereine, Goylette F Chami

**Affiliations:** Big Data Institute, Nuffield Department of Health, University of Oxford, Oxford, United Kingdom; Big Data Institute, Nuffield Department of Health, University of Oxford, Oxford, United Kingdom; Division of Vector Borne Diseases and Neglected Tropical Diseases, Uganda Ministry of Health, Kampala, Uganda; Division of Vector Borne Diseases and Neglected Tropical Diseases, Uganda Ministry of Health, Kampala, Uganda; Division of Vector Borne Diseases and Neglected Tropical Diseases, Uganda Ministry of Health, Kampala, Uganda; Division of Vector Borne Diseases and Neglected Tropical Diseases, Uganda Ministry of Health, Kampala, Uganda; Division of Vector Borne Diseases and Neglected Tropical Diseases, Uganda Ministry of Health, Kampala, Uganda; Big Data Institute, Nuffield Department of Health, University of Oxford, Oxford, United Kingdom

**Keywords:** gut inflammation, calprotectin, schistosomiasis

## Abstract

**Background:**

The role of gut inflammation for intestinal schistosomiasis remains poorly understood in chronically infected and repeatedly treated populations.

**Methods:**

We conducted a cross-sectional study nested in the SchistoTrack cohort within Pakwach district, Uganda. In 2024, 640 participants aged 6–85 years were examined for *Schistosoma mansoni* by Kato-Katz. Fecal calprotectin (fCal) concentration was measured by enzyme-linked immunosorbent assays. Fecal calprotectin was analyzed as binary outcomes (detectable, ≥100 µg/g, >250 µg/g) and natural log-transformed continuous values. Co-endemic infections (malaria, HIV, hepatitis B [HBV]) and sociodemographic covariates were investigated in logistic regressions with covariate selection.

**Results:**

74.4% of participants had detectable fCal, 22.3% had fCal ≥100 µg/g, and 7% had fCal >250 µg/g. *Schistosoma mansoni* prevalence was 49.1%. Infection intensity was positively associated with all fCal outcomes (detectable: OR 1.20, ≥100 µg/g: OR 1.11, >250 µg/g: OR 1.26; continuous: β .06) while infection status was positively related to all but the continuous fCal outcome. HIV was associated with fCal ≥100 µg/g (OR 2.52), while malaria and HBV were uninformative.

**Conclusions:**

*Schistosoma mansoni* infections are characterized by persistent, clinically concerning levels of gut inflammation in chronically infected populations with repeated praziquantel treatment. Integration of fCal thresholds into clinical guidelines may improve management of schistosomiasis-related morbidity.

## Teaser

*Schistosoma mansoni* infections are associated with clinically relevant fecal calprotectin thresholds. These associations remain despite chronic exposure and re-infection in endemic communities, a long history of mass drug administration with praziquantel, and the presence of other endemic infections.

Intestinal schistosomiasis is a complex disease or set of conditions, where *Schistosoma mansoni* is the main causative species in sub-Saharan Africa [1]. In endemic areas with high levels of schistosome re-exposure, chronic cases with a complex set of conditions of varying severity are observed. The most severe form is hepatosplenic schistosomiasis that includes periportal fibrosis (PPF) with hypersplenism, and ultimately complications of portal hypertension [2]. Less severe chronic presentations have been described with a focus on the nonspecific outcomes of anemia and diarrhea [1]. The presence and persistence of chronic gut inflammation is less understood, and there remains a frequent misconception that repeatedly infected individuals are asymptomatic [3].

Parasite survival depends on successful egg translocation through the intestinal mucosa into the stool, where they are released into the environment to continue the life cycle within intermediate snail hosts found in freshwater [1]. Granulomas are necessary for this passage; however, uncontrolled immune responses and microulcerations caused by egg-induced tears in the intestinal epithelium may lead to localized gut inflammation [4]. Microulcerations caused by egg translocations may induce bleeding, and in severe cases, potential anemia [5]. Yet, despite these less severe pathologies, the integrity of the gut lining is largely maintained, as there are few clinical cases of sepsis [5]—hypothesized to be due to the antimicrobial properties of schistosomes [6].

Fecal calprotectin (fCal) is a stable, nonspecific biomarker of intestinal inflammation, widely used in the preliminary diagnosis and monitoring of clinically relevant (ie requiring treatment) gut inflammation in inflammatory bowel diseases (IBD) such as Crohn's disease and ulcerative colitis [7–9]. It is a calcium-binding protein released primarily by neutrophils and macrophages in response to local inflammation. Elevated fCal levels have been positively associated with a range of factors, including viral [10], parasitic [11], and bacterial [12] infections. In schistosomiasis-endemic settings such as Uganda, there is high co-endemicity of malaria, human immunodeficiency virus (HIV), and hepatitis B virus (HBV) [2]. Each has been independently linked to gut inflammation through different, and sometimes overlapping, hypothesized pathways, such as uncontrolled viral replication (HIV) [13], microvascular sequestration of parasitized red blood cells in the gut (malaria) [14], and changes to the gut microbiota (malaria, HIV, and HBV) [10, 14, 15]. Findings from the limited number of schistosomiasis-specific studies on fCal [11, 16, 17] primarily focus on young children, do not account for the influence of co-infections, and show inconsistent associations. Important questions remain regarding the persistence of gut inflammation in endemic populations with high re-infection, and the relevance in the context of repeated mass drug administration (MDA).

In this study, we explored the relationship between *S. mansoni* infection with fCal levels within the community-based SchistoTrack cohort [18]. Our primary aim was to identify whether gut inflammation occurs within the context of chronically and repeatedly infected *S. mansoni* populations with a repeated history of praziquantel treatment through MDA, and whether this relationship is attenuated by co-infections.

## METHODS

### Study Context and Participant Sampling

**Figure 1.**
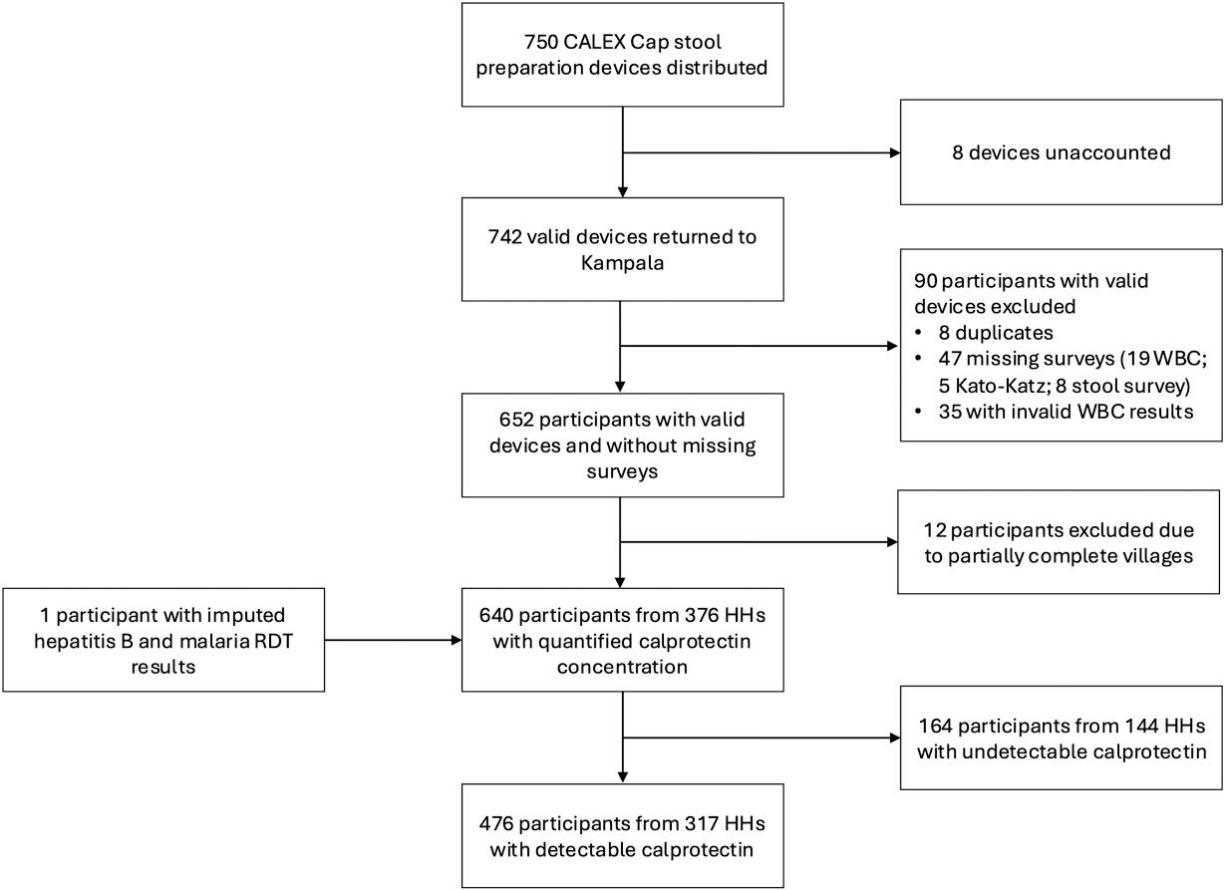
Participant selection. In 2024, the first 750 participants seen in Pakwach district within the existing SchistoTrack cohort were sampled. The flow diagram shows the completeness of the data and exclusion criteria to arrive at the 640 participants who were the focus of the study. A minimal detectable effect size of 11.1% for fCal prevalence between *S. mansoni* infected versus uninfected was possible with a sample of 640 individuals, and estimated *S. mansoni* prevalence of 43.3% and fCal prevalence of 50%, and 80% power for a 2-sided test. With the same assumptions, a minimal detectable effect size of 16.73% was possible at 99% power.

A nested, cross-sectional study was conducted in Pakwach district within the SchistoTrack cohort, where >50% *S. mansoni* prevalence has been observed [2, 18, 19]. SchistoTrack examines liver disease progression and related multimorbidity with annual follow-ups since 2022. As of 2024, Pakwach had at least fifteen annual rounds of school-based and community-based MDA. At recruitment for SchistoTrack, 1 child aged 5 years or older and 1 adult aged 18 years or older were selected within each randomly sampled household. Detailed SchistoTrack sampling is described elsewhere [18]. In 2024, 750 individuals from the first 12 study villages visited in Pakwach were targeted to oversample the population needed for study; several exclusion criteria were applied that focused on the completeness of exposures. Figure 1 shows the participant selection procedure and sample size information.

### Outcomes

Fecal calprotectin concentration was measured using commercial enzyme-linked immunosorbent assays kits (Bühlmann Laboratories AG) as a continuous concentration in µg/g of stool (Supplementary Methods). Three fCal outcomes were constructed for analysis. First, a binary outcome was defined as detectable fCal and coded as one if the concentration was greater than or equal to the detection limit, otherwise zero. Calprotectin levels lower than 100 µg/g indicate low IBD risk, values between 100–250 µg/g indicate an intermediate risk, while values above 250 µg/g indicate high risk [8, 9]. To examine the clinical relevance of these thresholds for *S. mansoni* infection, 2 additional binary outcomes were created based on ≥100 µg/g and >250 thresholds. To explore the relevance of fCal for schistosomiasis without relying on predefined clinical cutoffs, fCal was treated as a continuous variable and natural log-transformed to approximate a normal distribution. Individuals with values below the detection limit were excluded for this outcome, as their exact values were unknown but possibly nonzero.

### Exposures

Exposures included *S. mansoni* infection status and intensity (primary), malaria status and parasite density, HIV-1/2 status, and HBV status and chronicity (secondary). To explore clinical, mechanistic and confounding relationships with infections and fCal, white blood cell (WBC) differentials, fecal occult blood (FOB), hemoglobin (Hb) counts, gastrointestinal symptoms and relevant medications were measured. People living with HIV (PLHIV) were followed up in 2025 to measure viral loads, though all other analyses were cross-sectional. Detailed definitions of exposures are provided in the Supplementary Methods. Descriptions of sociodemographic covariates are provided elsewhere [18].

### Statistical Analysis

Analyses were run in R (v4.2.1) [20]. Logistic regression models for binary fCal outcomes and linear regression model for the continuous natural log-transformed fCal outcome were specified through backwards stepwise model selection using the lowest Bayesian information criterion to produce parsimonious interpretable models [20]. *Schistosoma mansoni* infection intensity, malaria parasite density, HIV status and HBV status were core variables forced into all models. We also examined *S. mansoni* and malaria infection status in place of intensity and density measures. To investigate potential mediators of fCal, natural log-transformed neutrophil count (continuous) and FOB (binary) were added to the models. Detailed analyses are described in the Supplementary Methods.

## RESULTS

### Fecal Calprotectin Variation

**Figure 2.**
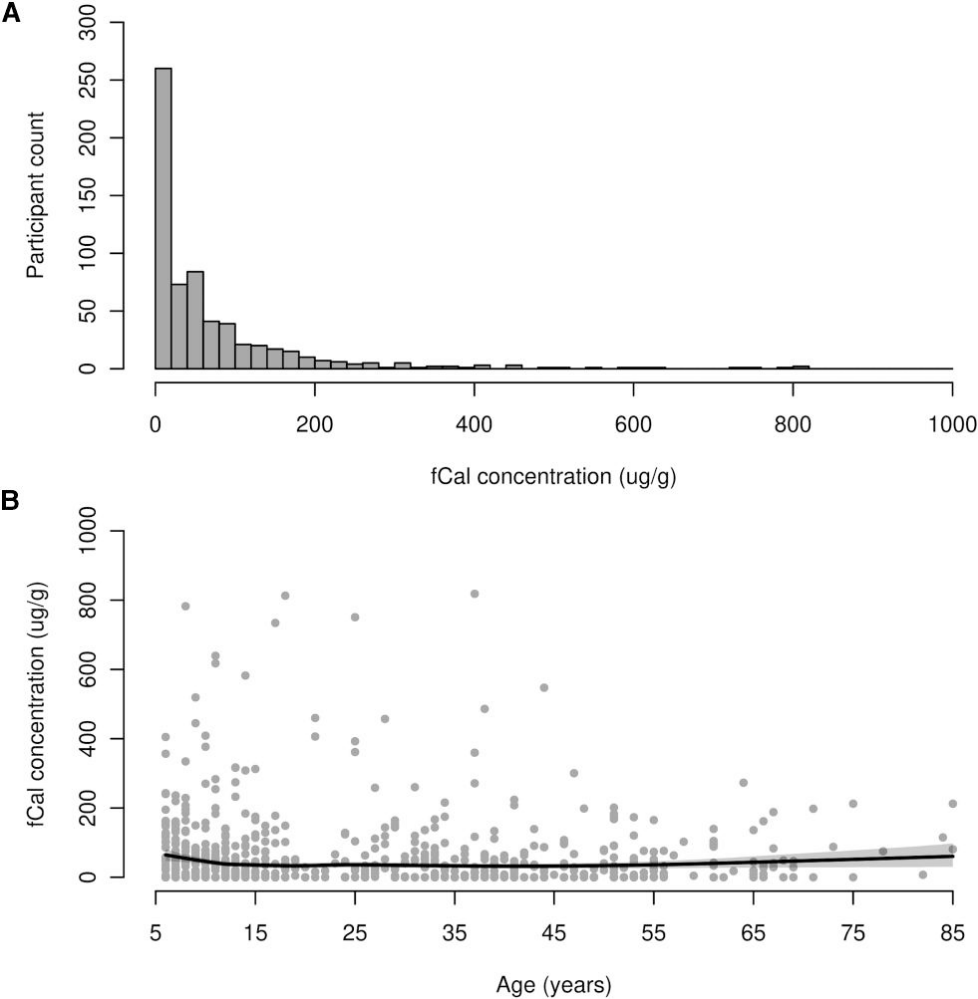
Calprotectin concentration among study participants (n = 631). *A*, Histogram of calprotectin concentration. *B*, Scatter plot of calprotectin concentration over age with a LOWESS curve (smoothing span = 0.5; black line with shaded areas representing bootstrap 95% CIs). Nine participants with calprotectin concentrations >1000 µg/g (range: 1490–2290 µg/g) were excluded from the plots.

Six hundred and forty of participants aged 6–85 from 376 households with complete data had fCal measured (Figure 1). Those participants were representative of the broader SchistoTrack cohort (Supplementary Table 1). 74.38% (476/640) of participants had detectable levels of fCal. The median detectable concentration was 54.77 µg/g (interquartile range 25.69–123.66). Fecal calprotectin had a left-skewed distribution (skewness 6.18, kurtosis 44.12) (Figure 2*A*). 22.34% (143/640) of participants (including those with undetectable fCal) had fCal concentration ≥100 µg/g, while 7.03% (45/640) had fCal concentration >250 µg/g. Across age, fCal was slightly elevated in young children (6–10 years) and in older adults (>60 years), although only 6.56% (42/640) of participants were over 60 (Figure 2*B*).

### Infection Prevalence

Participant characteristics are presented in Table 1. 49.06% (314/640) of the study participants were infected with *S. mansoni,* with 23.89% (75/314) of those infected classified as having high infection intensity (≥400 eggs per gram of feces (EPG)). 36.72% (235/640) of participants had malaria. 5.31% (34/640) of participants were HBV positive. Among the HBV positive participants, 85.29% (29/34) had been tested for HBV in 2023, of whom 75.86% (22/29) were chronically infected. 4.84% (31/640) of participants were living with HIV.

**Table 1.** Participant Characteristics

	Overall (N = 640)	Detectable fCal (N = 476)	Undetectable fCal (N = 164)
*S. mansoni* status (positive)^b^	314 (49.1%)	256 (53.8%)	58 (35.4%)
*S. mansoni* intensity (EPG)^a,b^	0 (0–144)	12 (0–192)	0 (0–24)
Malaria status (positive)^b^	235 (36.7%)	178 (37.4%)	57 (34.8%)
Malaria parasite density^a,b^	0 (0–0)	0 (0–3.9)	0 (0–0)
HIV status (positive)^b^	31 (4.8%)	25 (5.3%)	6 (3.7%)
HBV status (positive)^b^	34 (5.3%)	26 (5.5%)	8 (4.9%)
Age^a^	19 (11–40)	18 (10.75–39)	26 (11.75–43)
Gender (female)	328 (51.3%)	245 (51.5%)	83 (50.6%)
Occupation	…	…	…
Farmer	103 (16.1%)	79 (16.6%)	24 (14.6%)
Fisherman	30 (4.7%)	23 (4.8%)	7 (4.3%)
Fishmonger	6 (0.9%)	5 (1.1%)	1 (0.6%)
None/other	501 (78.3%)	369 (77.5%)	132 (80.5%)
Education	…	…	…
None	124 (19.4%)	94 (19.7%)	30 (18.3%)
Primary	471 (73.6%)	347 (72.9%)	124 (75.6%)
Secondary or above	45 (7.0%)	35 (7.4%)	10 (6.1%)
Year of recruitment	…	…	…
2024	64 (10.0%)	46 (9.7%)	18 (11.0%)
2023	470 (73.4%)	329 (69.1%)	141 (86.0%)
2022	106 (16.6%)	101 (21.2%)	5 (3.0%)
No. of people in household^a^	3 (3–4)	3 (3–4)	3 (3–4)
Home quality score^a^	3 (3–3)	3 (3–3)	3 (3–3)
Social status	108 (16.9%)	80 (16.8%)	28 (17.1%)
Home ownership	586 (91.6%)	431 (90.5%)	155 (94.5%)
Years lived in village^a^	17.5 (8–34.2)	17.5 (8–35)	17.5 (7.8–33.3)
Dist. to the nearest Gov’t health center (km)^a^	6.4 (5.2–12.2)	6.4 (3.9–12.2)	6.6 (5.8–12.1)
Main type of health facility used	…	…	…
Private clinic or other	43 (6.7%)	37 (7.8%)	6 (3.7%)
Gov’t health center	597 (93.3%)	439 (92.2%)	158 (96.3%)
Improved drinking water source	331 (51.7%)	218 (45.8%)	113 (68.9%)
Improved sanitation facility at home	307 (48.0%)	249 (52.3%)	58 (35.4%)
Household treated drinking water	173 (27.0%)	136 (28.6%)	37 (22.6%)
Any intestinal symptoms	251 (39.2%)	184 (38.7%)	67 (40.9%)
Any abdominal pain	199 (31.1%)	138 (29.0%)	61 (37.2%)
Any abdominal enlargement	20 (3.1%)	17 (3.6%)	3 (1.8%)
Vomiting or nausea	17 (2.7%)	15 (3.2%)	2 (1.2%)
Fecal occult blood	128 (20.0%)	101 (21.2%)	27 (16.5%)
Any blood in stool	50 (7.8%)	44 (9.2%)	6 (3.7%)
Blood in stool with diarrhea	23 (3.6%)	21 (4.4%)	2 (1.2%)
Blood in stool without diarrhea	33 (5.2%)	30 (6.3%)	3 (1.8%)
Melena	5 (0.8%)	4 (0.8%)	1 (0.6%)
Any diarrhea without blood	185 (28.9%)	143 (30.0%)	42 (25.6%)
Diarrhea (reported)	37 (5.8%)	33 (6.9%)	4 (2.4%)
Diarrhea (Bristol stool scale)	159 (24.8%)	120 (25.2%)	39 (23.8%)
Constipation (Bristol stool scale)	64 (10.0%)	48 (10.1%)	16 (9.8%)
Anemia	371 (58.0%)	267 (56.1%)	104 (63.4%)
Hemoglobin count (g/dL) (1 missing)^a^	11.4 (9.8–13.1)	11.6 (9.8–13.2)	11.3 (9.8–13.0)
Praziquantel *Past y*ear	519 (81.1%)	386 (81.1%)	133 (81.1%)
MDA	25 (3.9%)	16 (3.4%)	9 (5.5%)
Study	514 (80.3%)	384 (80.7%)	130 (79.3%)
Other deworming medications *Past month*	55 (8.6%)	40 (8.4%)	15 (9.1%)
Antimalarials *Past month*	202 (31.6%)	143 (30.0%)	59 (36.0%)
Antibiotics *Past month*	294 (45.9%)	217 (45.6%)	77 (47.0%)
Anti-inflammatories *Past month*	37 (5.8%)	22 (4.6%)	15 (9.1%)
ART *Ever*	26 (4.1%)	22 (4.6%)	4 (2.4%)
HBV ART *Ever*	0 (0.0%)	0 (0.0%)	0 (0.0%)
Total WBC count^a^	5.9 (4.5–7.53)	6.15 (4.7–7.73)	5.3 (4.1–6.7)
Neutrophil count^a^	2.1 (1.5–2.9)	2.15 (1.6–3)	1.9 (1.4–2.6)
Monocyte count^a^	0.4 (0.3–0.5)	0.4 (0.3–0.5)	0.3 (0.2–0.4)
Lymphocyte count^a^	2.9 (2.1–3.7)	3 (2.2–3.9)	2.7 (2–3.4)
Eosinophil count^a^	0.3 (0.1–0.6)	0.3 (0.1–0.6)	0.3 (0.1–0.5)
Basophil count^a^	0 (0–0)	0 (0–0)	0 (0–0)
Neutrophil-to-lymphocyte ratio^a^(2 missing)	0.73 (0.55–0.96)	0.74 (0.55–0.96)	0.70 (0.54–0.97)

Binary data are presented as N (%).

^a^Count or continuous data are presented as median (interquartile range [IQR]). WBC counts are in units of 10^9^.

^b^Among *S. mansoni*-positive participants, the median EPG was 144 (IQR 36–369), and the co-infection prevalence was 5.10% (16/314) for HBV, 3.18% (10/314) for , and 44.90% (141/314) for malaria. One participant was co-infected with HBV and . 83.87% (26/31) of PLHIV were aware of their status, and all of those (100%, 26/26) were currently on ART. Among malaria-positive participants by RDT, 61.28% (144/235) were also positive by microscopy with 96.53% (139/144) with species information; 89.93% (125/139) were *Plasmodium falciparum* only, 8.63% (12/139) were *P. falciparum* and *Plasmodium malariae* with 2 infections of *P. malariae* only. The median parasite density was 48 parasites/µL of blood (IQR 0–336; obs. 235).

### Determinants of Fecal Calprotectin

**Figure 3.**
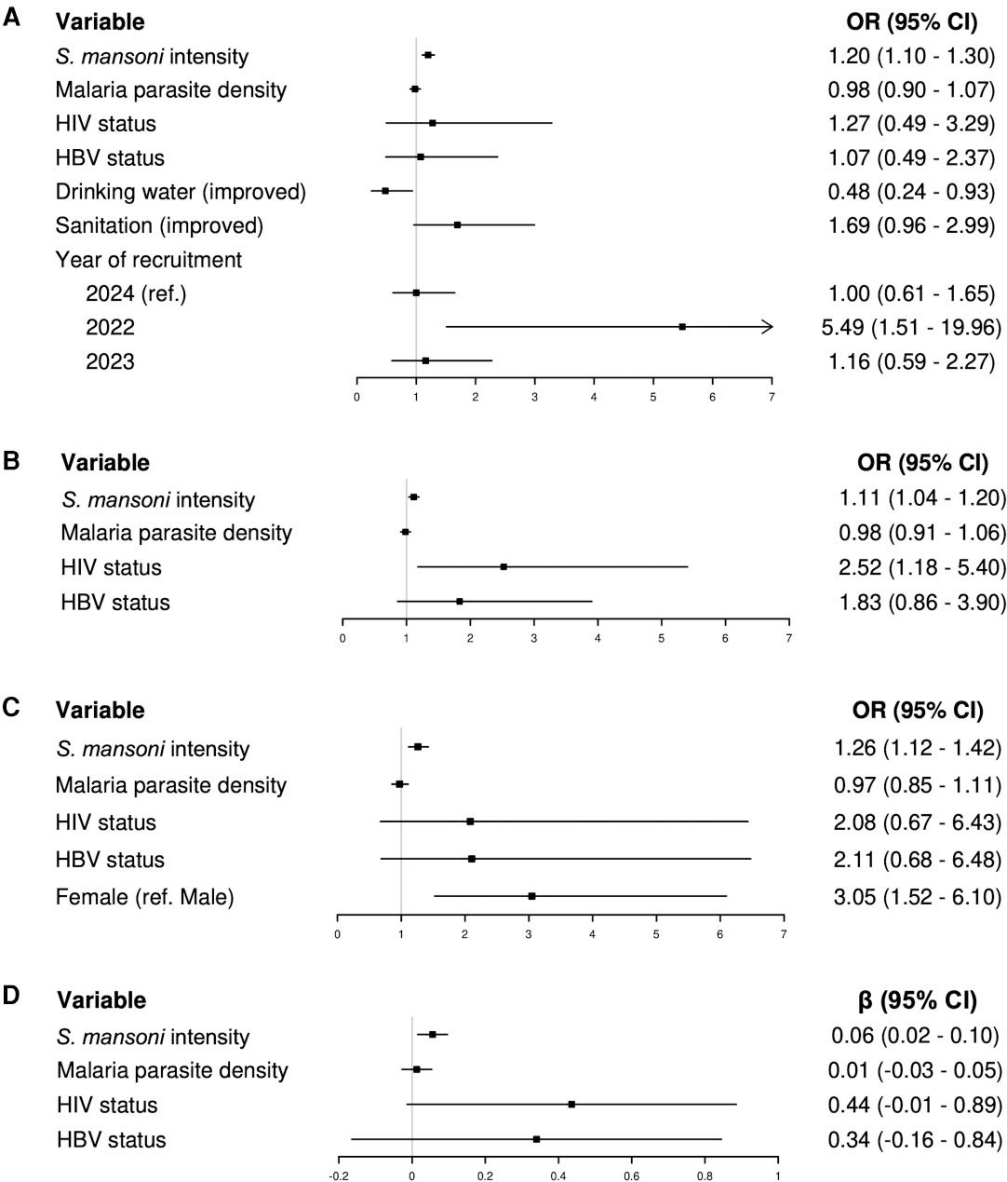
Calprotectin (fCal) models for infection intensity. Model of (*A*) detectable fCal, (*B*) ≥ 100 µg/g, (*C*) fCal >250 µg/g, and (*D*) linear fCal (N = 640). Logistic regression models were selected by backward stepwise selection based on the lowest Bayesian Information Criterion. Ninety-five percent CIs were calculated using clustered standard errors at the village level (for detectable fCal; number of villages = 12) and the household level (for linear fCal; number of households = 376). Floating absolute risks were calculated for the year of recruitment variable. Intraclass correlation coefficient (ICC) (village) = 0.147; 0.029; 0.014; 0.016. ICC (household) = 0.119; 0.062; 0.084; 0.102. Variance inflation factor (VIF) range: 1.00–1.09; 1.00–1.04; 1.01–1.14; 1.00–1.04. Area under the curve (AUC) for stratified 10-fold cross-validation = 0.69; 0.57; 0.70. R^2^ (linear model) = 0.03.

Comparison of participants with detectable and undetectable fCal are shown in Table 1. Unadjusted analyses are shown in Supplementary Tables 2–5. *Schistosoma mansoni* infection intensity was positively associated with all fCal outcomes in adjusted models (Figure 3). Each 2.72-fold increase in EPG was associated with 1.20 times higher odds of having detectable fCal (95% CI 1.10–1.30) and 1.11 times higher odds of fCal ≥100 µg/g (95% CI 1.04–1.20). The association was stronger for a higher fCal threshold of >250 µg/g (OR 1.26, 95% CI 1.12–1.42). In a linear model, a 10% increase in EPG was associated with a 0.57% increase in fCal (95% CI .19–.96%). HIV status was associated with 2.52 (95% CI 1.18–5.40) times higher odds of fCal ≥100 µg/g, although unrelated to high levels of gut inflammation (fCal >250 µg/g) (Figure 3*B* and 3*C*). Co-infection with HIV and *S. mansoni* was positively correlated with fCal ≥100 µg/g (Supplementary Figure 1). Malaria and HBV were not significantly associated with any fCal outcomes. Chronic HBV infection also was not correlated with any fCal outcomes (Supplementary Figure 1). Female participants had 3.05 (95% CI 1.52–6.10) times higher odds of fCal >250 µg/g than male participants; age was not selected in any fCal model (Figure 3). Access to an improved drinking water source was associated with 0.48 (95% CI .24–.93) times lower odds of having detectable fCal compared with unimproved source (Figure 3*A*). Improved sanitation facility and year of recruitment were selected in the model but not significant (Figure 3*A*). When *S. mansoni* infection intensity and malaria parasite density were replaced with infection status, *S. mansoni* associations remained significant for detectable fCal and low (≥100 µg/g) and high (>250 µg/g) inflammation levels, but were borderline insignificant for linear fCal (Supplementary Figure 2). Results remained robust when 1 participant with imputed malaria and HBV status was excluded from the models.

### White Blood Cells Differentials and fCal Production

**Figure 4.**
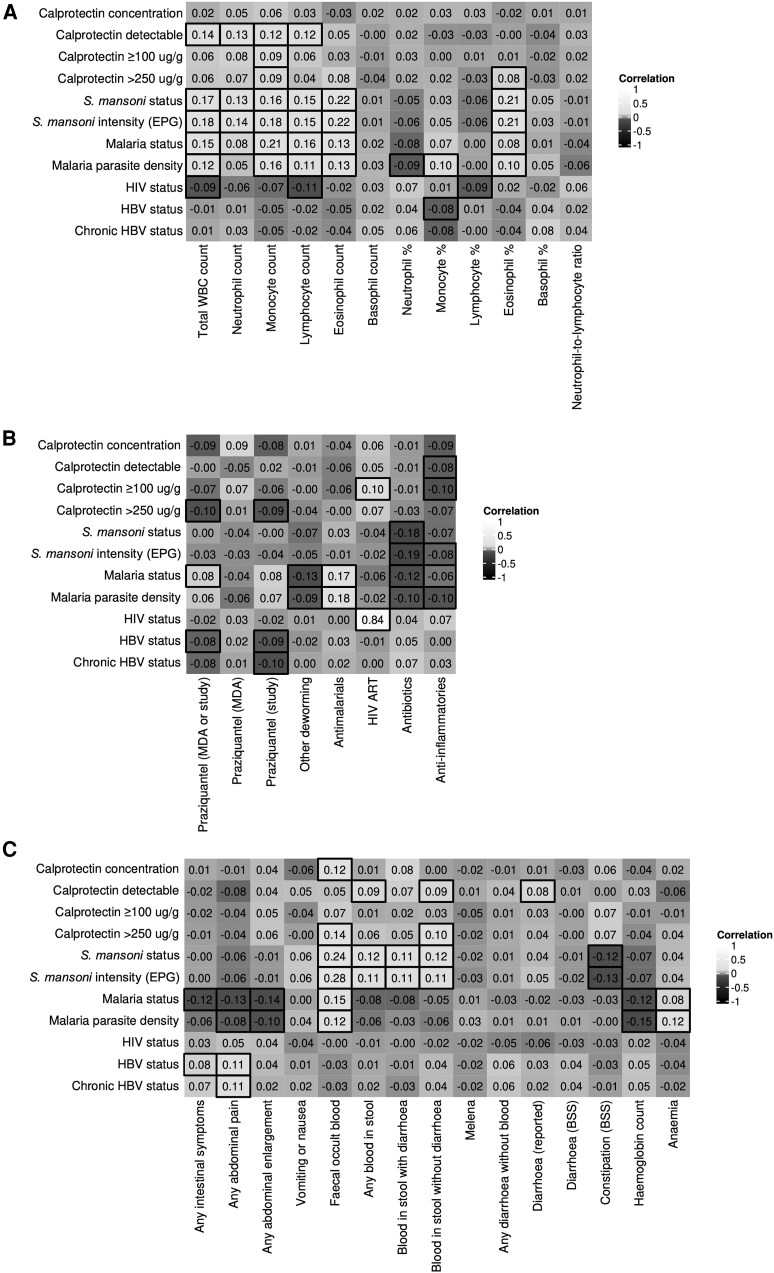
Spearman correlation matrices of (*A*) white blood cell differentials, (*B*) medications, and (*C*) symptoms with fCal outcomes and infections (*S. mansoni*, malaria, HIV and HBV). Black boxes represent correlations that were significant to *P* value < .05.

Total WBC, neutrophil and lymphocyte count were weakly positively correlated with detectable fCal (*r*_s_ 0.14, *P* < .05; *r*_s_ 0.13, *P* < .05; *r*_s_ 0.12, *P* < .05) (Figure 4*A*). Monocyte count was weakly positively correlated with detectable fCal and both fCal thresholds (*r*_s_ 0.12, *P* < .05 for detectable fCal; *r*_s_ 0.09, *P* < .05 for fCal ≥100 µg/g; *r*_s_ 0.09, *P* < .05 for fCal >250 µg/g), while eosinophil percentage was positively correlated only with fCal >250 µg/g (*r*_s_ 0.08, *P* < .05). All WBC differentials except for basophils were weakly to moderately positively correlated with *S. mansoni* infection intensity and status (*r*_s_ range 0.13–0.22, *P* < .05). The neutrophil-to-lymphocyte ratio was not significantly correlated with fCal or *S. mansoni* outcomes (*r*_s_ range −0.06–0.06, *P* ≥ .05). When added to the adjusted models, the natural logarithm of the neutrophil count was associated with 1.96 (95% CI 1.13–3.40) times higher odds of having detectable fCal only, where *S. mansoni* infection intensity and status remained significant (Supplementary Figures 3, 4).

### Medications as Potential fCal Confounders

Praziquantel taken within the past year (through MDA or the study) was negatively correlated only with high levels of gut inflammation (fCal >250 µg/g) (*r*_s_ −0.10, *P* < .05) (Figure 4*B*). Praziquantel slightly attenuated the effect of *S. mansoni* status in the adjusted model (OR 1.99, 95% CI 1.01–3.95), while it was not significant in the fCal models with *S. mansoni* intensity (Supplementary Figures 2C, 3C). Ever taking ART for HIV was positively correlated with fCal ≥100 µg/g (*r*_s_ 0.10, *P* < .05) (Figure 4*B*), but nonsignificant when added to the adjusted model (Figure 3*B*). 83.87% (26/31) of PLHIV had viral load measured, of which only 50% (13/26) were virally suppressed. Among PLHIV only, viral suppression was not significantly correlated with fCal. Taking anti-inflammatory medications was negatively correlated only with detectable fCal (*r*_s_ −0.08, *P* < .05) and fCal ≥100 µg/g (*r*_s_ −0.10, *P* < .05). Anti-inflammatory medications also were negatively correlated with *S. mansoni* infection intensity (*r*_s_ −0.08, *P* < .05), while antibiotics were negatively correlated with *S. mansoni* infection intensity (*r*_s_ −0.19, *P* < .05) and status (*r*_s_ −0.18, *P* < .05) but not fCal outcomes. In the adjusted models, anti-inflammatory medications were associated with 0.18 (95% CI .04–.77) times lower odds of fCal ≥100 µg/g, and a 31.61% decrease in fCal levels in the linear model (95% CI −51.32% to −2.96%) (Figure 3*B* and 3*D*). *Schistosoma mansoni* infection intensity remained significant in both models. *Schistosoma mansoni* status was no longer significant in the fCal ≥100 µg/g model, while it remained nonsignificant in the linear model. Anti-inflammatory medications were associated with 0.56 (95% CI .34–.93) times lower odds of having detectable fCal when added to infection status models only (Figure 3*A*), where *S. mansoni* status remained significant.

### Microulcerations Detected by Fecal Occult Blood and Gastrointestinal Symptoms

Fecal occult blood was weakly positively correlated with fCal concentration (*r*_s_ 0.12, *P* < .05) and fCal >250 µg/g (*r*_s_ 0.14, *P* < .05) (Figure 4*C*). Fecal occult blood also was moderately positively correlated with both *S. mansoni* infection intensity (*r*_s_ 0.28, *P* < .05) and status (*r*_s_ 0.24, *P* < .05). When added to the adjusted models, FOB was associated with 2.96 (95% CI 1.48–5.94) times higher odds of fCal >250 µg/, and a 0.30% increase in fCal concentration in the linear model (95% CI .08–.53%) (Supplementary Figure 5). *Schistosoma mansoni* infection intensity remained significant in the fCal >250 µg/g model, while it became borderline nonsignificant in the linear model (Supplementary Figure 5). *Schistosoma mansoni* status was insignificant in both FOB models (Supplementary Figure 6).

Having any blood in stool (with or without diarrhea) within the past month was weakly positively correlated with detectable fCal only (*r*_s_ 0.09, *P* < .05), while having blood in stool without diarrhea was weakly positively correlated with detectable fCal (*r*_s_ 0.09, *P* < .05) and fCal >250 µg/g (*r*_s_ 0.10, *P* < .05) (Figure 4*C*). Both symptoms were weakly positively correlated with *S. mansoni* infection intensity (*r*_s_ 0.11, *P* < .05; *r*_s_ 0.11, *P* < .05) and status (*r*_s_ 0.12, *P* < .05; *r*_s_ 0.12, *P* < .05). Having blood in stool with diarrhea within the past month was weakly positively correlated with *S. mansoni* infection intensity (*r*_s_ 0.11, *P* < .05) and status (*r*_s_ 0.11, *P* < .05) but not fCal outcomes. Reported diarrhea within the past month was positively correlated with detectable fCal only (*r*_s_ 0.08, *P* < .05), while constipation based on biosample analysis was negatively correlated with *S. mansoni* intensity (*r*_s_ −0.13, *P* < .05) and status (*r*_s_ −0.12, *P* < .05) but not fCal outcomes. Hb count and anemia were not correlated with fCal outcomes or *S. mansoni* infection intensity and status (*r*_s_ range −0.07–0.04, *P* ≥ .05). Only 1 participant reported vomiting blood.

## DISCUSSION

No World Health Organization guidelines based on clinical biomarkers exist for schistosomiasis morbidity. Here we investigated the relevance of fCal—a clinically established, noninvasive marker of gut inflammation—for *S. mansoni* infection, while considering co-endemic HIV, HBV, and malaria infections, alongside WBC differentials, medications, and symptoms. In 640 individuals aged 6–85 years from the community-based SchistoTrack cohort, *S. mansoni* infection status and intensity were consistently associated with elevated fCal levels, with evidence of contributing microulcerations.

*Schistosoma mansoni* infection—both intensity and status—were positively associated with fCal outcomes of varying severity, despite our study area having received fifteen rounds of annual MDA. Infection intensity was significant for all fCal outcomes, while infection status was significant for all outcomes except for linear fCal, where it was borderline nonsignificant. These findings support the potential relevance of fCal as an inflammatory biomarker for *S. mansoni*, even when applying established clinical thresholds for IBD, where fCal ≥100 µg/g indicates intermediate risk and fCal >250 µg/g indicates high risk of IBD [8]. Additional research is needed to assess the value of the fCal thresholds prospectively with schistosomiasis-related morbidity outcomes.

The association between *S. mansoni* status and gut inflammation, given chronic exposure in our study population, suggests that a high level of morbidity may persist in current infection. Surprisingly, age was not relevant and female gender was positively associated with fCal >250 µg/g, despite prior studies focusing exclusively on children (though not accounting for co-infections) and finding no associations with gender [11, 16, 17]. These findings raise the possibility that gut inflammation continues even in the presence of acquired immunity from repeated schistosome exposure (either due to age or gendered water contact activities) [21]. More detailed immunological investigations of the gut, such as proteomic analyses, are needed, alongside further epidemiological studies to understand the relationship between gut inflammation and sepsis beyond murine models [5].

Praziquantel treatment was negatively associated with high levels of gut inflammation (fCal >250 µg/g) and very minimally attenuated the effect of *S. mansoni* status on fCal. Notably, praziquantel had no significant effect on the relationship between infection intensity and gut inflammation. This suggests that although praziquantel effectively reduces infection intensity, it may not resolve established intestinal inflammation—likely because it has limited or varied effect on intact granulomas. Evidence from a murine model [22] supports this interpretation, showing that the impact of praziquantel on hepatic and intestinal granuloma volume depends on both dose and treatment duration, with shorter or single high-dose regimens being associated with smaller or no reductions.

Fecal occult blood showed a positive correlation with both fCal and *S. mansoni* and slightly attenuated the effect of *S. mansoni* intensity. Microulcerations and associated granulomatous inflammation in the gut epithelium may be caused by *S. mansoni* egg translocation and entrapment, and could be a major factor in clinically relevant gut inflammation [23]. Neither fCal or *S. mansoni* were correlated with anemia or hemoglobin count, suggesting a lack of severe complications due to intestinal bleeding, and questioning the use of conventional symptoms for schistosomiasis morbidity representation. Our findings extend beyond previous studies that have only linked FOB to schistosome infection [16, 17].

Higher neutrophil counts were linked to detectable fCal and *S. mansoni*, while monocytes were associated with all fCal outcomes and *S. mansoni*. Neutrophils act as early responders in acute inflammation, releasing calprotectin to recruit other immune cells, including monocytes [24]. Their contribution to chronic inflammation is emerging and may involve sustained immune activation and tissue damage [25]. The exact role of neutrophils in host immune response to *S. mansoni* infection is not well understood [26], as research has predominantly focused on eosinophils (also positively associated in our study). Our findings suggest circulating neutrophils contribute to gut inflammation in *S. mansoni* infection and highlight WBC differentials as useful indicators of immune response. Further research is needed to establish the clinical relevance of neutrophilia in schistosomiasis and compare circulating with gut-localized WBCs. Neutrophils also may reflect fCal release in response to acute inflammation from other infections. Future studies should focus on the correlation of WBCs with clinical outcomes such as PPF due to intestinal schistosomiasis.

In our study, HIV status was positively associated with fCal ≥100 µg/g. Although effective ART achieves viral suppression and improves life expectancy of PLHIV, morbidity remains due to chronic immune activation and inflammation [27]. The gut is a primary site of HIV replication, which leads to localized gut inflammation (a hallmark of HIV infection) [27]. Subsequent systemic inflammation and immune activation persist at residual levels even with successful ART [10, 28]. The independent associations of *S. mansoni* and HIV with fCal, and the preliminary positive correlation between schistosome-HIV co-infection (though few observations) and fCal, underscores the need to investigate a possible contribution of co-infections (including past schistosome infections) to gut inflammation, and to adjust for HIV as an independent confounder to schistosomiasis-related gut inflammation. Recent research has shown that elevated fCal in ART-treated PLHIV is linked to systemic inflammation, suggesting an important area for future research on schistosome-HIV co-infections [10].

Self-reported symptoms are frequently used for assessing acute *S. mansoni* infection [1]. In our study, blood in stool was weakly positively correlated with both fCal and *S. mansoni* infection, possibly indicating intestinal blood loss due to microulcerations [23]. *Schistosoma mansoni*, despite representing chronic infection or repeated exposure within our study population, remained (weakly) associated with a wide range of gastrointestinal symptoms, suggesting that symptoms should be used not only in acute but also chronic infection monitoring for schistosomiasis. In frequently re-exposed populations, chronic morbidity and the associated disability-adjusted life years may be underestimated if clinical manifestations are not fully captured in health states or disability weights [29–31]. Our finding that gut inflammation persists in repeatedly treated populations raises questions about how ongoing subclinical morbidity contributes to severe *S. mansoni*-associated morbidities, including end-stage PPF [2].

The strengths of this study lie in the comprehensiveness of co-infections, symptoms, and potential confounders observed across a wide age range of individuals. Concerning limitations, we did not collect data on concurrent protozoa, bacterial, and viral gut infections, which may influence fCal levels [12]. However, we attempted to partially account for those by incorporating higher-level environmental factors: improved sanitation facility, improved drinking water source, and household treated drinking water. These factors were not selected in our models, although individual-level hygiene behavior data were unavailable. Future work should directly measure gut pathogens as well as other causes of increased fCal including celiac disease, infectious colitis, and necrotizing enterocolitis. Although soil-transmitted helminths were not observed in our study district, they should be accounted for (especially hookworm) if co-endemic with schistosomiasis in other studies. Laboratory analyses were performed using a single sensitivity threshold with single-replicate testing, potentially limiting measurement precision. Data were collected at a single time point, providing only a cross-sectional snapshot. Kato-Katz microscopy may miss schistosome infections with very low or no egg output, though the clinical and mechanistic relevance of egg-negative infections is unclear. Future studies investigating such cases should consider adding circulating anodic antigen tests.

We showed that intestinal schistosomiasis is characterized by gut inflammation, particularly in chronically infected populations where significant morbidity due to *S. mansoni* persists despite repeated MDA. The observed link between *S. mansoni* infection and elevated fCal supports its potential role as a practical, noninvasive biomarker for gut inflammation in intestinal schistosomiasis. Incorporating such measures to develop schistosomiasis morbidity or clinical management guidelines could improve patient management within primary healthcare facilities beyond controlling infection alone.

## Supplementary Material

jiag094_Supplementary_Data

## Supplementary Data

Supplementary materials are available at *The Journal of Infectious Diseases* online (http://jid.oxfordjournals.org/). Supplementary materials consist of data provided by the author that are published to benefit the reader. The posted materials are not copyedited. The contents of all supplementary data are the sole responsibility of the authors. Questions or messages regarding errors should be addressed to the author.
